# Experimental *Ixodes ricinus*-Sheep Cycle of *Anaplasma phagocytophilum* NV2Os Propagated in Tick Cell Cultures

**DOI:** 10.3389/fvets.2020.00040

**Published:** 2020-02-06

**Authors:** Consuelo Almazán, Lisa Fourniol, Clotilde Rouxel, Pilar Alberdi, Christelle Gandoin, Anne-Claire Lagrée, Henri-Jean Boulouis, José de la Fuente, Sarah I. Bonnet

**Affiliations:** ^1^UMR BIPAR, INRAE, Ecole Nationale Vétérinaire d'Alfort, ANSES, Université Paris-Est, Maisons-Alfort, France; ^2^SaBio, Instituto de Investigación en Recursos Cinegéticos IREC (CSIC-UCLM-JCCM), Ciudad Real, Spain; ^3^Department of Veterinary Pathobiology, Center for Veterinary Health Sciences, Oklahoma State University, Stillwater, OK, United States

**Keywords:** *Anaplasma phagocytophilum*, *Ixodes ricinus*, sheep, NV2Os, tick cell cultures

## Abstract

The causative agent of tick-borne fever and human granulocytic anaplasmosis, *Anaplasma phagocytophilum*, is transmitted by *Ixodes ricinus*, and is currently considered an emerging disease throughout Europe. In this study, we established a model of *A. phagocytophilum* sheep infection and *I. ricinus* transmission using the European Norway variant 2 ovine strain (NV2Os) propagated in both IDE8 and ISE6 tick cells. Two sheep were inoculated with IDE8 tick cells infected with NV2Os. Both sheep developed *A. phagocytophilum* infection as determined by qPCR and PCR, the presence of fever 4 days post inoculation (dpi), the observation of morulae in granulocytes at 6 dpi, and the detection of *A. phagocytophilum* antibodies at 14 dpi. *A. phagocytophilum* was detected by PCR in skin, lung, small intestine, liver, spleen, uterus, bone marrow, and mesenteric lymph node from necropsies performed at 14 and 15 dpi. One sheep was infested during the acute phase of infection with *I. ricinus* nymphs from a pathogen-free colony. After molting, *A. phagocytophilum* transstadial transmission in ticks was validated with qPCR positive bacterial detection in 80% of salivary glands and 90% of midguts from female adults. Infected sheep blood collected at 14 dpi was demonstrated to be able to infect ISE6 tick cells, thus enabling the infection of two additional naive sheep, which then went on to develop similar clinical signs to the sheep infected previously. One of the sheep remained persistently infected until 115 dpi when it was euthanized, and transmitted bacteria to 70 and 2.7% of nymphs engorged as larvae during the acute and persistent infection stages, respectively. We then demonstrated that these infected nymphs were able to transmit the bacteria to one of two other naive infested sheep. As expected, when *I. ricinus* females were engorged during the acute phase of infection, no *A. phagocytophilum* transovarial transmission was detected. The development of this new experimental model will facilitate future research on this tick-borne bacterium of increasing importance, and enable the evaluation of any new tick/transmission control strategies.

## Introduction

*Anaplasma phagocytophilum* (Rickettsiales: Anaplasmataceae), is an obligate intracellular Gram negative bacterium mainly transmitted by *I. scapularis* and *I. pacificus* in the United States, *I. persulcatus* in Asia, and *I. ricinus* in Europe (1). *A. phagocytophilum* is the causative agent of human granulocytic anaplasmosis (HGA) and tick-borne fever (TBF), affecting both humans and a variety of domestic and wild animal species (2–4). Tick-borne fever was first identified in sheep from Scotland in 1932 (5), and the discovery of the etiological disease agent followed in 1940 (6). Throughout Europe, sheep are exposed to *A. phagocytophilum* (7)—with seroprevalence as high as 80% in sheep grazing in tick-infested Norwegian pastures—resulting in considerable economic and animal welfare consequences (8, 9).

The wide range of potential hosts, as well as incidence and severity of the disease in a particular host appear to vary according to geographical region (1). *A. phagocytophilum* infects granulocytes, mostly neutrophils, and exists as macrocolonies or morulae within intracytoplasmic vacuole (10, 11). Approximatively within a week of exposure to an infectious tick bite, TBF disease becomes clinically evident and is characterized by fever, leukopenia, marked neutropenia, and thrombocytopenia (12). In sheep, *A. phagocytophilum* infection causes a fever lasting from 1 to 2 weeks, and which may vary according to animal age, the *A. phagocytophilum* variant, the host breed and its immunological status (13). Anorexia, depression (14), reduced weight gain (15), as well as abortions (16) have also been reported. In addition, because of the severe hematological and immune disorders associated with *A. phagocytophilum* infection, animals are more susceptible to secondary infections, including tick pyemia, caused by *Staphylococcus* spp. (14).

*A. phagocytophilum* has a large number of genetic variants which vary in virulence and clinical manifestation, and which can be differentiated by sequencing 16S rRNA (9) or *mps4* genes (17, 18). Four variants that differ in pathogenicity and immunogenicity were identified as circulating in sheep flocks from Norway (9). Among them, *A. phagocytophilum* variant 1 (var1) is believed to be associated with the majority of fatal TBF cases in sheep (19). In contrast, *A. phagocytophilum* variant 2 (var2), which is also frequently found circulating in naturally-infected sheep, produces a less severe clinical manifestation with shorter periods of fever and bacteremia, and a less severe neutropenia (20–22). Indeed, as it produces mild TBF in sheep, *A. phagocytophilum* var2 could be a useful model for studying bacterial infection processes in sheep, tick transmission modalities, and could also be used in vaccine trials.

Tick cell lines are well-established systems in which tick-borne pathogens—including *Anaplasma* species—can be propagated (23). Both HGA and equine granulocytic anaplasmosis variants were initially cultivated in the human promyelocytic cell line HL-60 (24), and then in IDE8 and ISE6 tick cell lines derived from *I. scapularis* embryos (25, 26). TBF variants were successfully cultivated in these tick cell lines, including variants isolated from sheep, such as the Old Sourhope strain, which, after three culture passages, was shown to be infectious in susceptible sheep (27). The *A. phagocytophilum* Norway var2 ovine strain (NV2Os), isolated from Norwegian sheep (9), was also successfully propagated in IDE8 embryonic tick cells (28), but, to the best of our knowledge, sheep infection from this cell culture has never been experimentally achieved to date.

The present study aims to investigate whether the *A. phagocytophilum* NV2Os propagated in both IDE8 and ISE6 tick cells can be used in the laboratory to experimentally recreate the entire bacterial transmission cycle from sheep to ticks and from ticks to sheep, thus creating an experimental model in which both tick-host-pathogen interactions, as well as novel tick/transmission control strategies, such as anti-tick or anti-transmission vaccines, can be studied. We report that the development of this model enabled successful sheep infection, the production of infected *I. ricinus* from both acute and persistently *A. phagocytophilum*-infected sheep, as well as the re-transmission of the bacteria to naïve sheep by these ticks.

## Materials and Methods

### Experimental Design

**Figure 1:**
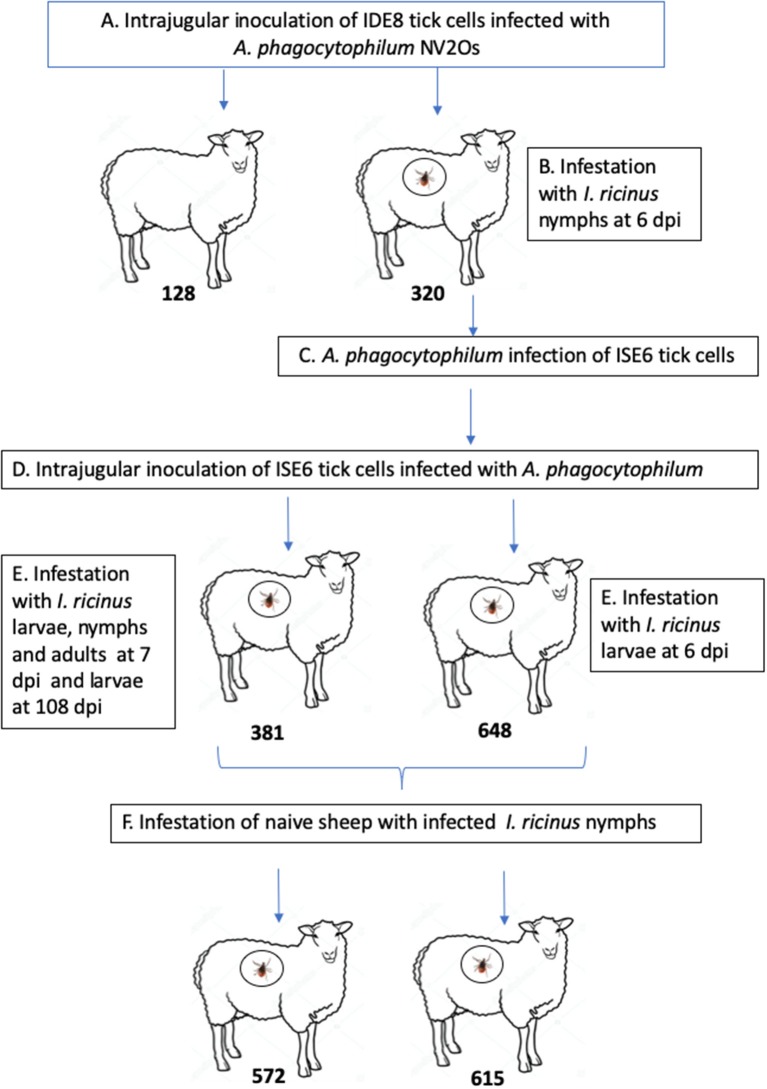
Schematic representation of the experimental design. **(A)** Infection of Romane sheep (identification numbers 128 and 320) with *Anaplasma phagocytophilum* NV2Os propagated in IDE8 tick cells; **(B)** *Ixodes ricinus* infestation; **(C)** Infection of ISE6 tick cells with the *A. phagocytophilum* NV2Os from sheep 320; **(D)** Inoculation of Romane sheep (identification numbers 381 and 648) with the *A. phagocytophilum* NV2Os propagated in ISE6 tick cells; **(E)** Transmission of *A. phagocytophilum* from sheep to *I. ricinus* ticks during both acute (sheep 381 and 648) and persistent infection (sheep 381); **(F)** Tick transmission of *A. phagocytophilum* to naive PreAlps sheep (identification numbers 572 and 615).

The experimental design of the study is presented in Figure 1. Two 9-month-old Romane breed female sheep (identification numbers 128 and 320) were inoculated with IDE8 tick cells infected with *A. phagocytophilum* NV2Os into the jugular vein, and euthanized at 14 and 15 days post inoculation (dpi), due to clinical signs of distress. During the acute phase of *A. phagocytophilum* infection at 6 dpi, sheep 320 was infested with *I. ricinus* nymphs. In addition, infected blood obtained from sheep 320 was used to assess whether NV2Os could be propagated in ISE6 tick cells. These *A*. *phagocytophilum*-infected ISE6 tick cells were then intrajugularly inoculated into two 11-month-old Romane breed female sheep (identification numbers 381 and 648). Unexpectedly, sheep 648 died at 12 dpi, and no necropsy could be performed. Sheep 381 however, recovered after the acute phase and remained without signs of infection until 115 dpi, when it was euthanized. *A*. *phagocytophilum* tick transmission was evaluated by infesting both sheep 381 and 648 during the acute phase of infection at 7 and 6 dpi, respectively, as well as sheep 381 during the persistent phase of infection at 108 dpi with *I. ricinus* larvae, and the infection assessed after molting into nymphal stages. The infectious status of nymphs molted from larvae fed on infected sheep 381 and 648 was assessed by feeding them on two naive PreAlps breed sheep (identification numbers 572 and 615). For sheep 128 and 320, blood samples were obtained every day from day 0 to 14 and 15 dpi, respectively. Sheep 648 was bled every day from day 0 until 12 dpi, when it died. Sheep 381 was bled every day from day 0 to 15 dpi, and then every 15 days until 115 dpi. Sheep infected with *I. ricinus* nymphs (572 and 615) were bled every 5 days from day 0 to day 25 post tick infestation.

### Culture of *A. phagocytophilum* NV2Os in IDE8 and ISE6 Tick Cells

IDE8 and ISE6 embryonic tick cell cultures were maintained according to Munderloh and Kurtti (29) and Munderloh et al. (30), respectively. Healthy tick cells from both cell lines were propagated in L-15B medium, whereas infected cells were cultured in L-15B supplemented with 0.1% NaHCO_3_ and 10 mM HEPES and the pH was adjusted to 7.5. Both uninfected and infected IDE8 and ISE6 cells were maintained at 31 and 34°C, respectively. The *A. phagocytophilum* NV2Os was propagated in IDE8 tick cells (approximate passage of 110) as described by Alberdi et al. (28). *A. phagocytophilum* infection was propagated by transferring 1/10th of an infected IDE8 cell culture to a new flask of healthy cells, every 4 days. To determine the level of infection, 60 μl of RPMI medium with suspended cells were centrifuged on slides with a cytocentrifuge Shandon Cytospin (Thermo Fisher Scientific, Sweden) for 5 min at 1,000 rpm, stained using the Hemacolor® staining kit (Merck, Darmstadt, Germany) and observed under a light microscope.

Blood from *A. phagocytophilum*-infected sheep 320 collected at 14 dpi was used to infect ISE6 tick cells. A 500 μl drop of blood was added to two 25 cm^2^ culture flasks of ISE6 cells (approximate passage of 90). Cytocentrifuged slides were stained twice a week using Hemacolor® kit (Merck Millipore, Darmstadt, Germany) in order to determine the level of infection. Once infection reached 70%, cells (~1 × 10^7^ infected cells) were collected and centrifuged for 5 min at 200 × g, then resuspended in 2 ml of sterile RPMI to prepare the inoculum (31).

### Sheep Inoculation

Four 9-month-old Romane breed female sheep, originally from the INRA Experimental Animal Center at Bressonvilliers, France, and reared in a secure sheepfold at the Biomedical Research Center (CRBM) facilities, National School of Veterinary Medicine of Alfort (ENVA), were used. First, two sheep identified with numbers 128 and 320 (Figure 1) were inoculated with 1 ml of medium containing 1 × 10^7^ *A. phagocytophilum*-infected IDE8 cells with a 70% infection level into the jugular vein with a 6 cc syringe and a 20-gauge 1” needle (Terumo) after skin disinfection. Two months later, the two other sheep, with identification numbers 381 and 648 (Figure 1) were inoculated with 1 ml of medium containing 5 × 10^6^ ISE6 cells infected with *A. phagocytophilum* obtained from sheep 320 and with a 70% infection level.

### Detection of *A. phagocytophilum* in Sheep's Blood by PCR and Determination of Infection Levels by Quantitative PCR (qPCR)

PCR detection of *A. phagocytophilum msp4* was performed using DNA obtained from blood collected daily, from day 0 to 14 or 15 dpi in sheep inoculated with IDE8 infected cells (sheep 320 and 128, respectively), and from day 0 to 12 or 15 dpi in sheep inoculated with ISE6 infected cells (sheep 648 and 381, respectively). For sheep infected by *I. ricinus* nymphs (572, 615), PCR detection was performed every 5 days from 0 to 25 days post tick infestation and until euthanasia. Blood was collected from the jugular vein, and drawn into 10 ml EDTA vacutainer tubes. The PCR protocol described by Kocan et al. was followed (32). Briefly, DNA was extracted using a NucleoSpin Blood kit (Macherey-Nagel, Germany) from 200 μl of blood. PCR reactions were then performed using the oligonucleotide primers msp4-F (5′-CCTTGGCTGCAGCACCACCTG-3′), and msp4-R (5′-TGCTGTGGGTCGTGACGCG-3′), in 20 μl of final volume using the Takara Ex Taq system (Bio Europe, France). For positive and negative controls, DNA from *A. phagocytophilum-*infected ISE6 tick cells and nuclease free water were used, respectively. PCR products were visualized by 2% agarose gel electrophoresis. PCR products were purified using the PCR Clean-Up kit (Macherey Nagel, Germany), and were sequenced by the Eurofins sequencing service (France). The obtained sequences were submitted to the BLAST (basic local alignment search tool) platform to search for sequences with homology to *A. phagocytophilum* NV2Os.

*A. phagocytophilum* infection levels in sheep were determined by qPCR targeting the *msp4* gene using DNA obtained from blood collected every 3 days from sheep 320 and 128, starting at day 0 until euthanasia (14 and 15 dpi, respectively), and from sheep 648 and 381 until 12 and 15 dpi respectively. For sheep 381, qPCR was performed every 15 days until 115 dpi. Samples were processed according to the protocol described by Reppert et al. (31). DNA concentration was evaluated with a NanoDrop™ 2000 (Thermo Scientific, USA) and 20 ng of DNA was then mixed in a 20 μl reaction containing the primers qmsp4F (5-ATGAATTACAGAGAATTGCTTGTAGG-3), and qmsp4R (5-TTAATTGAAAGCAAATCTTGCTCCTATG-3) using the SsoAdvanced™ SYBR® Green Supermix (Bio-Rad, Hercules, CA, USA). Reactions were performed in a LightCycler® 480 (Roche Life Science, Indianapolis, IN, USA). The *Ovis aries* aldolase B gene (*ALDOB*) was used for normalization, with the primers Oa-AldBF (5′-CCCATCTTGCTATCCAGGAA-3′) and Oa-AldBR (5′-TACAGCAGCCAGGACCTTCT-3′). The same mix without DNA, or DNA from infected ISE6 cell culture were used as negative and positive controls, respectively. Triplicate values from each sample were normalized by calculating the ratio of *A. phagocytophilum msp4* DNA to the averaged *ALDOB* gene. The standard errors of the averaged normalized values of the mean were determined and values analyzed by the Student's *t*-test (*p* = 0.05).

### Detection of *A. phagocytophilum* Intracellular Inclusions in Blood Smears

Blood smears were performed on blood samples collected daily in EDTA tubes, starting at day 0 until 14 dpi for sheep 320, 15 dpi for sheep 128 and 381, and 12 dpi for sheep 648. Blood smears were stained using the Hemacolor® staining kit (Merck, Darmstadt, Germany). Slides were observed under a light microscope and 100 white cells per slide were examined to determine the percentage of infected neutrophils. When basophilic inclusions consistent with *A. phagocytophilum* organisms were found, slides were then examined under an Imager 21 Zeiss microscope adapted to an AxioCam HRC Zeiss camera to record images.

### Gross Lesions and Detection of *A. phagocytophilum* in Sheep Tissues

In order to detect gross lesions, necropsies were performed immediately after euthanasia, which was carried out via intravenous injection of 18% sodium pentobarbital. Euthanasia occurred at 14 and 15 dpi for sheep 320 and 128 during the acute phase of the infection, and at day 115 for sheep 381 during the persistent phase of the infection. For PCR detection of the *A. phagocytophilum msp4* gene, ~1 cm^2^ of skin from the tick infestation area, and samples from sheep lung, myocardium, liver, spleen, stomach, small intestine, lymph nodes, brain, uterus, ovary, cerebellum, bone marrow, kidney, and gallbladder were obtained, deposited into sterile tubes and stored at −80°C until later use. DNA was extracted using a NucleoSpin® tissue kit (Macherey-Nagel, Germany) from 25 mg of each tissue. DNA concentration was evaluated with a NanoDrop™ 2000 (Thermo Scientific, USA) and PCR reactions were performed using 20 ng of DNA in a final volume of 20 μl as described for blood in section Detection of *A. phagocytophilum* in Sheep's Blood by PCR and Determination of Infection Levels by Quantitative PCR (qPCR).

### Indirect Immunofluorescence Antibody Assay (IFA)

The detection of anti-*A. phagocytophilum* antibodies was performed using the indirect immunofluorescence antibody assay (IFA) on serum samples obtained at day 0 (for negative controls), and at 14 dpi and 15 dpi from sheep 320 and 128, respectively; and every 15 days until 115 dpi for sheep 381. Blood samples were collected from the jugular vein, and serum obtained by centrifugation. Serial dilutions of serum starting from 1:50 were analyzed using the Mega-Screen Fluo *A. phagocytophilum-*coated slides (MegaCor, Hoerbranz, Austria), following manufacturer's instructions. Briefly, the *A. phagocytophilum-*coated slides were incubated with serial dilutions of serum for 30 min at 37°C. Slides were rinsed with PBS and incubated with 25 μl of 1:50 fluorescein isothiocyanate-conjugated rabbit anti-sheep IgG (H + L) (Jackson Immuno Research, Cambridge, UK) in Evans blue solution (Biomerieux, Hampshire, UK) for counterstaining. After incubation for 30 min at 37°C, slides were rinsed and mounting medium (Bio-Rad, Hercules, CA, USA) was added. Finally, slides were analyzed with a fluorescent microscope. A titer of 1.69 (Log_10_ reciprocal of 1:50) or higher was considered positive (8).

### Tick Infestation

*I. ricinus* ticks originally collected from the Sénart Forest, France (coordinates 48°40′00″N 2°29′00″E), and maintained as a pathogen-free colony reared at 22°C with 95% relative humidity and a 12-h light/dark cycle as previously described (33), were used for sheep infestation. For feeding, ticks were enclosed into stockinet cells attached to the back of each sheep, following a protocol adapted from cattle and rabbits (34, 35). Nine hundred nymphs of *I. ricinus* were engorged on sheep 320 when *A. phagocytophilum* was detected by PCR, during the highest fever spike (6 dpi) (Figure 1). Engorged nymphs were collected after 6 days of feeding. Collected engorged ticks were cleaned and reared until they molted into adults. The transmission of *A. phagocytophilum* to *I. ricinus* ticks during acute infection of sheep was tested by infesting 950 naive nymphs on sheep 381 and 2,000 naive larvae on sheep 381 and 648 at 7 and 6 dpi, respectively (Figure 1). Engorged larvae were collected after 3–5 days of feeding and then incubated in order to test for *A. phagocytophilum* presence in both engorged ticks and recently molted nymphs. Transovarial transmission of *A. phagocytophilum* was tested by infesting 15 adult couples on sheep 381 at 7 dpi. Engorged females were collected after 9 days of feeding and reared until oviposition. The transmission of *A. phagocytophilum* to *I. ricinus* ticks during persistent infection of sheep was tested by infesting 4,000 naive larvae on sheep 381 at 108 dpi (Figure 1). Finally, in order to test if nymphs molted from larvae fed on *A. phagocytophilum*-infected sheep 381 and 648 were able to transmit the bacteria to naive sheep, two 8-month-old male PreAlps breed sheep, identified as 572 and 615, were infested with 45 nymphs each (Figure 1).

### Detection of *A. phagocytophilum* in Ticks

*A. phagocytophilum* detection by qPCR was performed using DNA obtained from salivary glands and midguts from adult ticks engorged as nymphs on infected sheep 320, whole nymphs engorged as larvae on sheep 381 and 648, as well as egg masses obtained from females engorged on sheep 381 (Figure 1). DNA was extracted from each individual tick with the NucleoSpin tissue kit (Macherey Nagel, Germany), and 20 ng of DNA was mixed in a 20 μl reaction containing the primers msp2-F (5-ATGGAAGGTAGTGTTGGTTATGGTATT-3), and msp2-R (5- TTGGTCTTGAAGCGCTCGTA), adapted from the protocol described by Courtney et al. (36), using the SsoAdvanced™ SYBR® Green Supermix (Bio-Rad, Hercules, CA, USA). Reactions were performed in triplicate in a LightCycler® 480 (Roche Life Science, Indianapolis, IN, USA). Normalization was performed using the tick ribosomal protein S4 gene (*rps4*) with primers rps4F 5- GGTGAAGAAGATTGTCAAGCAGAG-3 and rps4R 5- TGAAGCCAGCAGGGTAGTTTG-3 (37).

## Results

### *A. phagocytophilum* NV2Os Propagated in Both IDE8 and ISE6 Tick Cells Can Infect Sheep

**Figure 2:**
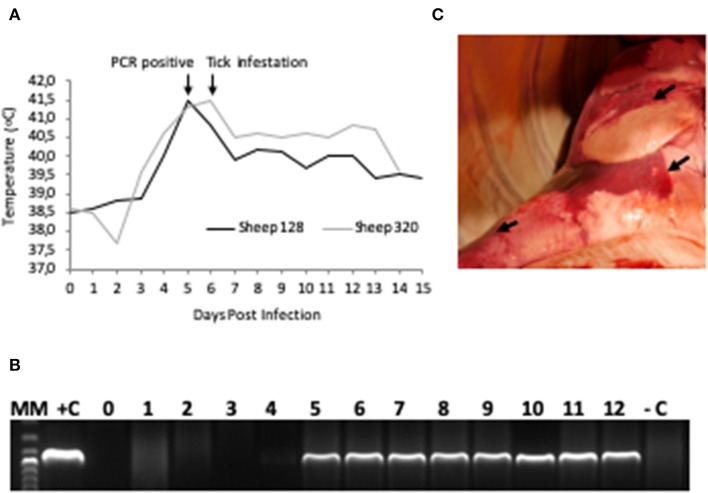
*A. phagocytophilum* infection in sheep experimentally infected with NV2O-infected IDE8 tick cells. **(A)** Temperature (°C) recorded daily from day 0 post-infection to the day of euthanasia for sheep 128 and 320; **(B)** PCR detection of *A. phagocytophilum msp4* gene in sheep 320 blood samples from day 0 to day 12 post-infection, MM, molecular marker; +C, positive control; –C, negative control; **(C)** Lung necropsy of sheep 320 14 days post infection. Arrows indicate patches of red coloration contrasting with the pink normal color.

**Figure 3:**
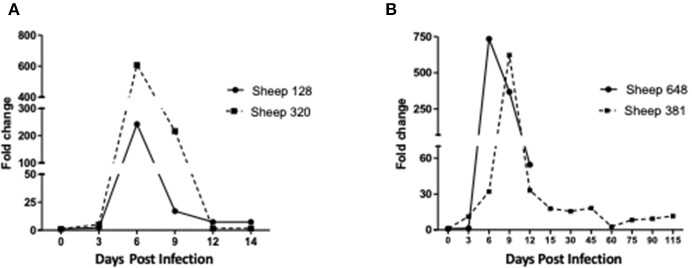
Infection levels in sheep inoculated with *A. phagocytophilum* NV2Os propagated in tick cells. Infection levels were determined from day 0 post-infection to the day of euthanasia and according to *A. phagocytophilum msp4* gene expression as assessed by quantitative PCR (qPCR) relative to the *aldolase B* (*ALDOB*) gene of *Ovis aries*. Triplicate values from each sample were normalized by calculating the ratio of *A. phagocytophilum msp4* DNA to the averaged *ALDOB* gene. **(A)** Sheep 128 and 320 inoculated with NV2Os cultivated in IDE8 tick cells; **(B)** Sheep 381 and 648 inoculated with NV2Os cultivated in ISE6 tick cells.

**Figure 4:**
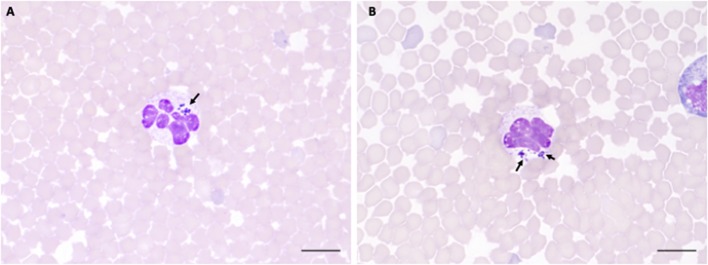
*A. phagocytophilum* morula (arrowheads) in peripheral blood neutrophils from sheep infected with NV2Os propagated in IDE8 tick cells. Blood smears were performed at day 8 post-infection and stained with the Hemacolor® staining kit (Merck). **(A)** Sheep 320; **(B)** Sheep 128. Scale bars = 10 μm.

The two sheep (128, 320) inoculated with the *A. phagocytophilum* NV2Os propagated in IDE8 tick cells developed an infection as demonstrated by clinical signs, observation of morulae inclusions in neutrophils, positive PCR and qPCR results, anti-*A. phagocytophilum* antibody detection, as well as the presence of lesions detected during necropsies after euthanasia. The first sign of infection was increased temperature by the 3rd dpi with peaks of 41.5°C at 5 and 6 dpi in sheep 128 and 320, respectively (Figure 2A). After 7 dpi, a decrease in appetite, and the presence of nasal discharge and conjunctivitis were observed in both animals. Sheep 320 maintained a temperature over 40°C until 13 dpi, at which point nasal discharge and lacrimal secretions increased, it then became lethargic with tachypnea, prostration, and stopped drinking water. Due to animal welfare regulations and ethics, euthanasia was performed at 14 and 15 dpi for sheep 320 and 128, respectively. *A. phagocytophilum msp4* PCR was positive from 5 dpi until 12 dpi in both sheep (Figure 2B). For both sheep, qPCR-determined *A. phagocytophilum* infection levels increased after 3 dpi, showed the highest fold change at 6 dpi, and then decreased drastically at 9 dpi. However, *A. phagocytophilum* DNA remained at detectable levels until 14 dpi in sheep 128 (Figure 3A). The statistical analysis of the averaged normalized values of the mean indicated that the fold change was significant (*p* = 0.05) from 3 dpi until 14 dpi. *A. phagocytophilum* morulae were found in neutrophils from both infected sheep at 6 and 7 dpi, whereas no other blood infection was detected (Figure 4). The highest cellular infection level was observed at 8 dpi with 47 and 60% of neutrophils infected in sheep 128 and 320, respectively (Figure 4). Few morulae were seen at 10 dpi and no further intracellular inclusions were found after 12 dpi.

The main gross lesions observed during necropsies of the two sheep 128 and 320 at 14 and 15 dpi corresponded to a moderate hepatomegaly and splenomegaly. The spleens from both sheep showed rounded borders and mild enlargement. While the use of barbiturates for euthanasia may have contributed to the observed mild splenomegaly, the absence of bleeding during necropsy suggests that it was mainly due to infection with *A. phagocytophilum*. In addition, in contrast to a normal pink lung color, patches of red coloration in sheep 320 lungs were observed (Figure 2C), as well as abundant bronchial and tracheal liquid. Mesenteric, axillar, and mandibular lymph nodes from both sheep were also mildly increased in size. Lesions were not detected in the remainder of examined tissues. *A. phagocytophilum msp4* PCR on necropsies yielded positive results in lung, liver, spleen, uterus, and the small intestine from sheep 128, while in sheep 320, skin, spleen, lung, small intestine, bone marrow, and mesenteric lymph nodes were positive (Table 1).

**Table 1:** PCR detection of *Anaplasma phagocytophilum* in necropsies obtained after euthanasia of sheep 320 and 128 infected with infected IDE8 cells, at 14 and 15 dpi, respectively, and of sheep 381 infected with ISE6 cells at 115 dpi.

**Sheep n^**°**^**	**Skin**	**Lungs**	**Myocardium**	**Liver**	**Spleen**	**Stomach**	**Kidney**	**Gallbladder**	**Small intestine (Duodenum)**	**Mesenteric lymph nodes**	**Axillary lymph nodes**	**Prescapular lymph nodes**	**Uterus**	**Ovary**	**Brain**	**Cerebellum**	**Bone marrow**
320	+	+	–	–	+	–	–	–	+	+	–	–	–	–	–	–	+
128	–	+	–	+	+	–	–	–	+	–	–	–	+	–	–	–	Not analyzed
381	–	–	–	–	–	–	–	–	–	–	–	–	–	–	–	–	–

The two sheep (381, 648) inoculated with *A. phagocytophilum* propagated in ISE6 tick cells showed clinical signs similar to sheep 128 and 320. Increased temperature was observed at 5 dpi with a 42°C peak at 6 dpi. Sheep 648 presented with other clinical signs, such as trembling, total loss of appetite, and excessive salivation, and died at 12 dpi. Sheep 381, on the other hand, recovered after the febrile process, without any clinical signs until 115 dpi, when it was euthanized. *A. phagocytophilum msp4* PCR was positive for both sheep at 5 dpi, and sheep 381 became negative at 15 dpi (data not shown). qPCR-Determined infection levels showed the highest fold change at 6 dpi in sheep 648, and at 9 dpi in sheep 381 followed by a drastic decrease at 12 dpi for both sheep (Figure 3B). For sheep 381, significant values (*p* = 0.05) were obtained until the end of the experiment, except at 60 dpi. Granular inclusions consistent with *A. phagocytophilum* morulae were detected in both sheep, with the highest percentages of infected neutrophils at 8 dpi with 47 and 60% in sheep 381 and 648, respectively. No other blood pathogens were identified via the blood smears from the two sheep, including sheep 648 that died at 12 dpi. For sheep 381, except for a mild splenomegaly, no gross lesion was observed at 115 dpi and all tested tissues were negative for *A. phagocytophilum* by PCR (Table 1).

Antibody detection by IFA indicated that the four sheep, 128, 320, 381, and 648 were negative prior to experimental inoculations (titer ≤ 1.6), and sheep 128, 320, and 381 became positive at 14 dpi (titer = 2.0). Sheep 648 died at 12 dpi before seroconversion, and sheep 381 remained positive with antibody titers between 2.0 and 2.77 from 14 to 115 dpi, until the animal was euthanized.

### Transmission of *A. phagocytophilum* NV2Os by *I. ricinus*

The percentages of engorged ticks collected after feeding on infected sheep, as well as the rate of *A. phagocytophilum* detection in molted ticks are shown in Table 2. On all sheep, 67.5 and 11.3% of larvae and nymphs underwent successful engorgement, respectively. On sheep 320 inoculated with *A. phagocytophilum*-infected IDE8 tick cells, 17.7% of tick nymphs became engorged, and 50% underwent molting. *A. phagocytophilum* transstadial transmission was demonstrated by qPCR in 8 salivary glands (80%) and 9 midgut samples (90%) obtained from 10 dissected females infected at the nymphal stage. For sheep 648 inoculated with *A. phagocytophilum*-infected ISE6 tick cells, 46.2% of tick larvae became engorged, and where no transstadial transmission was detected in 10 recently-molted nymphs engorged at the larval stage. However, when testing sheep 381—also infected with ISE6 cells—during both the acute (7 dpi) and persistent phases of infection (108 dpi), *A. phagocytophilum* acquisition and transstadial transmission from larvae to nymphs was demonstrated. In fact, at 7 dpi, 7 of the 10 recently-molted nymphs infected at the larval stage were positive for *A. phagocytophilum* infection, whereas at 108 dpi, only 2.7% (2/72) were positive despite an 85.3% larval engorgement success rate. At 7 dpi, only 4.5% of nymphs became engorged, with 34.9% of engorged nymphs then molting into adults. Following the engorgement of 15 pairs of adult ticks on infected sheep 381 at 7 dpi, *A. phagocytophilum* transovarial transmission was tested using DNA extracted from five egg masses obtained from engorged females, and all samples were negative (data not shown).

**Table 2:** *Ixodes ricinus* ticks collected after feeding on *A. phagocytophilum*-infected sheep during both the acute or the persistent phases of infection, and detection of infection in engorged and molted ticks.

**Sheep n^**°**^**	**Day post-infection**	**Number of infesting ticks**		**Number of engorged ticks (%)**		**Number of ticks recovered after molting (%)**		***A. phagocytophilum*****-positive molted ticks/tested ticks (%)**	
		**Larvae**	**Nymphs**	**Larvae**	**Nymphs**	**Nymphs**	**Adults**	**Nymphs**	**Adults**
320	6	–	900	–	160 (17.7%)	–	80 (50%) (37 females, 43 males)	–	8/10 Salivary glands (80%) 9/10 midgut (90%)
648	6	2000	–	925 (46.2%)	–	252 (27.2%)		0/10	–
381	7	2000	950	1060 (53%)	43 (4.5%)	411 (38.8%)	15 (34.9%) (9 females, 6 males)	7/10 (70%)	–
	108	4000	–	3414 (85.3%)	–	2236 (65.5%)	–	2/72 (2.8%)	–

Finally, the transmission of *A. phagocytophilum* by *I. ricinus* nymphs fed as larvae on infected sheep 381 and 648, was tested by infesting two PreAlps sheep (572 and 615) with 45 nymphs each. To achieve this, a mixed pool of nymphs from both sheep was used, leading to an estimated 42% infection level in each tick batch. Nymph engorgement was as low as 3/45 (7%) in sheep 572, and 10/45 (22%) in sheep 615. Although sheep 572 remained healthy and *A. phagocytophilum* was not detected, sheep 615 developed hyperthermia above 40°C with inappetence, lethargy, and reluctance to drink water, which led us to perform euthanasia at 15 days post nymph infestation. These clinical signs were then similar to what was previously observed during an NV2Os infection, and this infection was confirmed by positive qPCR bacterial detection in blood samples as early as the 5th day post tick infestation. Sequencing the PCR-amplified *msp4* gene demonstrated that the isolate corresponded to *A. phagocytophilum* NV2Os, Gene Bank accession number CP015376.1.

## Discussion

As the etiological agent causing both HGA in humans and TBF in domestic animals, *A. phagocytophilum* receives growing scientific interest due to its importance in public health, to livestock welfare, and national economies. In the United Kingdom, there are an estimated 300,000 cases of tick pyaemia each year caused by immunosuppression in *A. phagocytophilum*-infected animals (14). A similarly high number of *A. phagocytophilum*-infected lambs has been reported in Norway (8). For livestock exposed to tick-infested pastures, current control methods are based on the use of long-acting antibiotics—given before animals are moved from tick-free environments into tick-infested pasture—and by reducing tick infestation with acaricides. Due to the multiple disadvantages of such chemical control measures (development of resistance, environmental hazard, contamination of milk, and meat products with drug residues, high cost), new approaches are urgently needed. The development of such approaches requires the establishment of relevant laboratory models. Therefore, the purpose of the present study was to develop an *I. ricinus*-sheep model that enabled us to mimic the natural *A. phagocytophilum* infection cycles of both sheep and ticks. This would facilitate future studies on tick-host-pathogen interactions, as well as enabling the evaluation of new control methods, such as anti-tick vaccines or transmission-blocking vaccines. It is well-known that for both vertebrates and ticks, tick-borne pathogen acquisition differs between natural infection via tick bites and experimental infection through injection due to, among other reasons, saliva-assisted transmission mechanisms (38).

In this study, only a limited number of sheep were included for this first experiment which aimed to validate our experimental model. Therefore, infection follow-up results and clinical observations should be taken with caution, and should ideally be reproduced to take into account any individual sheep variation and be generalized to the NV2Os/Romane breed sheep pair.

It has been reported that the delay in detecting *A. phagocytophilum* in sheep may vary according to the infectious dose, bacterial genotypes, infection source (infected blood or cell culture), as well as to the immune status and susceptibility of animal breeds [see review in (1)]. Here we report for the first time that the Norway variant 2 ovine strain (NV2Os) propagated in both IDE8 and ISE6 tick cell cultures was able to successfully infect Romane breed female sheep. Following infection with NV2Os-infected IDE8 cells, the bacteria were detected in sheep blood at 4 dpi by qPCR, 5 dpi by conventional PCR and 6 dpi by microscopic examination of blood smears, which is consistent with the assumed sensitivity of these detection techniques. Such a prepatent period in sheep is shorter than what was reported with the human strain NY-18 (10–21 dpi) (31, 32), longer than with the var1 (13, 22), but in accordance with what was observed with var2 in Norwegian Dala breed lambs (22). According to qPCR results, bacteremia peaked at 6 dpi, except for sheep 381 that recovered after the acute phase with a peak at 9 dpi following infection with *A. phagocytophilum* NV2Os propagated in ISE6 cells. Nevertheless, blood smears demonstrated a detection lag, with the maximum number of infected neutrophils (47 and 60%) at 8 dpi for sheep infected with IDE8 cells, suggesting also that qPCR detection is more reliable. The observed initial bacteremia period with a mean duration of 10.75 (±1.7) days, as determined by qPCR, was also similar to that reported by Granquist et al. for the Var2 where the mean was 11.4 (±1.8) (22). Lastly, infection monitoring in sheep 381 and positive qPCR bacterial detection until 115 dpi confirmed that *A. phagocytophilum* can establish long-term infections in immune competent sheep, and has previously been reported for as long as 6–25 months (22, 39–41). Such persistent cyclic activity was suggested to be linked to variant-specific antigen immune responses to the bacteria (42, 43), and is epidemiologically very important in the maintenance of *A. phagocytophilum* infection in the field, especially during periods of no tick activity.

The indirect immunofluorescence assay, adapted to detect antibodies against *A*. *phagocytophilum*, and which has been extensively used in experimental and natural infections (8, 9, 21, 22) was used to test seroconversion in infected sheep. Our findings showed that antibodies were detected in all infected sheep at 14 dpi. Although the titers varied during the persistent infection of sheep 381, the results remained positive until 115 dpi, when the animal was euthanized. These results are consistent with studies in naturally- and experimentally-infected sheep (9, 13, 22).

In accordance with previous studies using the same strain but in Norwegian Dala sheep by Stuen et al. and Granquist et al. (21, 22), the first clinical sign was an increase in temperature up to 41.5°C between 3 and 6 dpi dependent on the sheep. However, a discrepancy was observed that may be explained by the genetic susceptibility of the French breeds used in this study, where the febrile process lasted between 8 and 9 days here and 2.2–3 days in their studies. All infected sheep also showed signs of loss of appetite and lethargy until euthanasia, except for sheep 381 that recovered after the acute phase of infection, and showed no clinical signs until euthanasia at 115 dpi.

Few studies have been performed in order to evaluate the existence of tissue that may act as *A. phagocytophilum* reservoirs in persistently-infected sheep and which could explain the maintenance of the bacteria in the field, especially during periods of vector tick non-activity. When the Suffolk breed was infected with the NY-18 human strain, no bacteria could be detected in any internal organ 1 month after infection, even with quantitative PCR (31). However, when the Norwegian Dala breed was infected with a Norway ovine field isolate, several organs (bone marrow, intestinal and bladder walls, kidney, lymph node, and thymus) were PCR positive 3 months after infection (44). Here we observed positive PCR results in several organs from sheep euthanized 2 weeks after the infection, but no infection was detected by PCR during necropsies performed at 115 days post infection. These varying results may reflect differences due to the bacterial strain or sheep breed, but the main conclusion is that more experiments with more animals are needed to answer this question. However, preferential involvement of lymphoid tissue may suggest that these tissues could represent a source of infected cells that, when released into the blood, would enable tick infection (44). As for the lesions*, A. phagocytophilum* infection has been reported to be associated with moderate tissue damage, which targets lymphoid tissues and the spleen in particular (31, 32, 45), as confirmed by our study.

Experimentally-infected sheep here proved to be excellent hosts for the production of *I. ricinus* ticks infected with ovine NV2Os, with feeding success rates of 67, 11, and 60% for larvae, nymphs, and female adults, respectively. Unlike laboratory animals, such as mice or rabbits, the large size of sheep makes it possible to feed many ticks at once without animal suffering in the absence of infection. This result is very encouraging considering the fact that in an experimental study with *I. scapularis*, Kocan et al. reported larvae feeding failure but, like us, poor performance of nymphs feeding on sheep (32). After engorgement during the acute phase, ticks acquired *A*. *phagocytophilum*, with an estimation of 80% acquisition in both engorged larvae and nymphs (data not shown). Similar *A. phagocytophilum* acquisition percentages in field populations of *I. ricinus* have been reported in a study performed in the United Kingdom. There, sheep naturally exposed to tick-infested pasture for a minimum of 4 weeks, resulted in 59 and 72% of PCR-positive engorged larvae and nymphs, respectively, after feeding on sheep. It must be taken into consideration however, that nymphs may have been previously infected (46).

In this last study, the authors reported that after molting, engorged larvae led to 18.5% of infected nymphs, where engorged nymphs led to 48% of infected adults (46). Here, higher transstadial infection rates were recovered after feeding larvae and nymphs on *A. phagocytophilum*-experimentally-infected sheep during the acute phase, with 70% of nymphs infected, and 80 and 90% of female tick salivary glands and midguts infected, respectively. The differences could be due to bacterial strain and sheep breed, and/or to the fact that in our study, ticks were fed during the acute phase of the infection, at the point where bacteria reached their highest peak in blood, as demonstrated by qPCR. Indeed, in the study performed by Ogden and coworkers, ticks were collected from sheep undergoing both acute and post-acute phases of infection at different time points over 9 weeks (46). In addition, it has been reported that tick acquisition efficiency gradually declines from high levels in the acute and immediate post-acute infection phases, to low levels in sheep that have been infected for much longer periods (47).

This last observation was confirmed by our results on transstadial transmission efficiency of *A. phagocytophilum* in *I. ricinus* ticks fed during acute or persistent infection of the sheep 381 with 70% and only 2.7% infection levels in molted nymphs, respectively. These last low infection rates can be explained by the low bacteremia in persistently-infected sheep, and the fact that larvae ingest small amounts of blood—and hence bacteria—within blood meals. Indeed, in nymphs or adults, which ingest larger volumes of blood and consequently have access to higher infectious bacterial doses, we can expect higher infection percentages. In addition, it has been suggested that a greater number of feeding adult ticks stimulates bacterial multiplication at the tick bite site and thus increases the *A. phagocytophilum* infectious dose for ticks (48, 49). Here, only larvae were used to infest sheep during the persistent infection, when both larvae and nymphs were used during the acute infection, which may also explain the observed difference in tick infection levels. However, bacterial transmission to ticks from persistently-infected sheep with no clinical signs, no positive PCR bacterial detection in blood, and for which all necropsies were PCR negative, has a real epidemiological importance for bacteria and disease maintenance and propagation in the field.

Our results also showed that larvae which had acquired *A. phagocytophilum* NV2Os from an infected sheep were able to retransmit it, as nymphs, to a naive sheep, thus validating that the transstadial transmitted bacteria remained infectious. This is the first demonstration of a complete NV2Os transmission cycle, from an *in vitro* bacterial culture to *I. ricinus* ticks. Finally, the failure to detect bacteria in eggs laid by adult ticks engorged on infected sheep during the acute phase, confirms that transovarial transmission of *A. phagocytophilum* is not likely to occur, in correlation with previous studies (32, 46).

## Conclusion

Here we established a sheep model of IDE8 and ISE6 tick cell culture-propagated *A*. *phagocytophilum* NV2Os infection and tick transmission successfully mimicking the entire transmission cycle in the laboratory. Previously, sheep were experimentally infected with *A*. *phagocytophilum* NV2Os by inoculating blood obtained from infected sheep, but to our knowledge, this is the first time that this strain propagated in IDE8 or ISE6 tick cells has been used to infect sheep, and which produced clinical signs due to *A. phagocytophilum* infection. Our results also showed that not only were infected sheep able to transmit the bacteria to ticks during the acute phase of the infection, but also during the chronic persistent infection phase, a finding which has important epidemiological significance. Moreover, we demonstrated that nymphs infected on sheep during the preceding infection stage were also able to transmit the bacteria to naive sheep. The establishment of such a model opens an entire spectrum of possibilities in which to study the molecular events of *A*. *phagocytophilum* infection in both vertebrate and invertebrate hosts, tick-host-pathogen interactions, as well as testing the efficacy of vaccine candidates against *I. ricinus* and *A*. *phagocytophilum*.

## Data Availability Statement

The datasets generated for this study are available on request to the corresponding author.

## Ethics Statement

The animal study was reviewed and approved by ComEth Anses/ENVA/UPEC; Permit Number 2016092716395004, ComEth Anses/ENVA/UPEC; Permit Number 2015081414257726.

## Author Contributions

CA and SB conceived and designed the study, analyzed the results, and wrote the paper. JF advised on experimental design. CA and LF performed animal experiments and performed laboratory analyses. H-JB, CA, and LF performed necropsies. PA and CR performed the cell cultures. CG performed IFA. PA, CR, and A-CL edited the manuscript. All authors read and approved the final paper.

### Conflict of Interest

The authors declare that the research was conducted in the absence of any commercial or financial relationships that could be construed as a potential conflict of interest.
